# Attitudes and Values of Physical Education Professionals and Undergraduate Students about Their Role in Health Promotion

**DOI:** 10.3390/ijerph17072288

**Published:** 2020-03-28

**Authors:** Júlio César Nasário, Victor Zaia, Camila Martins Trevisan, Simone Garzon, Antonio Simone Laganà, Erik Montagna

**Affiliations:** 1 Faculdade de Medicina do ABC, Centro Universitário Saúde ABC, 09060-870 Santo André, Brazil; julio@unidavi.edu.br (J.C.N.); victorzaia@gmail.com (V.Z.); camilatrevisan22@gmail.com (C.M.T.); erik_montagna@yahoo.com (E.M.); 2 Faculty of Physical Education, Centro Universitário para o Desenvolvimento do Alto Vale do Itajaí (UNIDAVI), 89160-93 Rio do Sul, Brazil; 3 Department of Obstetrics and Gynecology, “Filippo Del Ponte” Hospital, University of Insubria, 21100 Varese, Italy; simone.garzon@univr.it

**Keywords:** school physical education, physical education teacher, physical education curriculum, health promotion, health education, childhood obesity, lifelong physical activity

## Abstract

Physical education (PE) is identified with health, with PE teachers and school PE regarded as legitimate instruments for health promotion. The PE teacher’s conceptions, attitudes, and values regarding the role of PE are inseparable from their performance. Thus, the objective of the present work is to verify concepts and attitudes of PE professionals and undergraduate students, in order to verify how they value their role in health promotion. This was a cross-sectional study that used surveys to assess attitudes and values of PE professionals and undergraduate students about their concepts of the role of PE in health promotion. A total of 942 PE professionals and undergraduate students regards themselves as players in health promotion (86.9%) despite no clear definition about the concept of health or the curriculum to attain such a goal, mainly based on academic training only. Also, they attribute the responsibility for childhood obesity and lack of motivation for the practice of physical activity to external factors, such as media (72.6%), family (84.7%), and technologies (83.1%). Despite participants regarding themselves as players in health promotion, there is a loose definition on how to promote health, and how to provide curriculum and strategies to meet the needs of public health.

## 1. Introduction

Physical education (PE) is closely identified with health in school curricula nowadays, with the PE teacher playing a central role in its deployment and implementation [1]. The concept of health in these curricula range from a fitness-centered concept, with a closer relationship between physical activity and health [2], to a broader and even critical view with an exacerbated value about what health actually is [3]. 

Despite the many concepts and their variations, two concurrent views are main standpoints among the spectrum [4]. The so-called “critical perspective”, encompassing broader health and physical activity concepts, is less directed at load, repetition, and intensity of exercise, and also includes the called “salutogenic perspective”, where the individual develops his or her own health, considering the sociocultural background, as well as personal needs and self-perceptions as regulators of the motivation to move along the health–disease spectrum [3]. They are opposed to the called “biomedical perspective”, which is fitness-centered, focused on exercise and associating nutrition with physical activity as a way to achieve a healthy condition [5]. This concept admits that the individual will incorporate physical activity as a habit, focused on intensity and repetition, with the PE teacher mainly as an instructor who delivers a package of tasks to be performed. The curricula delivered are directed toward public health objectives, focused on developing lifelong physical activity [6]. In this sense, PE play a central role in health promotion by contributing to stimulating a lifelong commitment to physical activity as a preventive factor against sedentarism [7]. 

The effects of sedentarism are known to be a risk factor for many diseases. Among the consequences of a sedentary lifestyle, obesity is one of the most alarming, specially childhood obesity. Several implications for health in the short and long terms are reported and well known, leading to medical, psychological, and social problems [8]. It will be the cause of a series of diseases of high prevalence with enormous consequences for the general health of the individual, and significant social burden for both productive sector and health systems. It is reported that more than 276 million adolescents are overweight or suffer from obesity worldwide [9]. 

Several causes contribute to this complex scenario, among them an obesogenic environment that comprises energy imbalance related to food intake and a reduction of time expended with physical activity and sedentarism [10]. The situation is the same in Brazil. Recent data showed that about 8% of students aged 13 to 17 years were obese and 23.7% overweight, or 1 million and 3.1 million in absolute numbers, respectively [11]. As a result, the Brazilian government proposed goals and incentives in order to improve this situation, which included scholar PE as one of the potential fronts of intervention. The federal program, entitled “Health in the School” (*Programa Saúde na Escola*) [12], was launched in 2007, promoting several actions of prevention, promotion, and care in health, and clearly mentions the relation between physical activity and health, as well as the role of PE in the program.

Whatever the approach, the notion of health is the subject of extensive debates in the literature and has a series of considerations that are out of the scope of the present work [13]. In this sense, the notion of health adopted in the present work assumes a broader principle of health [14], not necessarily aligned with the biomedical or critical perspective of the PE. Besides that, we will assume that school PE is an legitimate instrument for health promotion, and is an adjuvant for increasing the level of physical activity [15]. Even in front of the undeniable consequences of physical inactivity for health, the practice of physical activity depends on the individual’s habits and attitudes towards engagement with physical activity and healthy habits [16]. Thus, among the strategies for stimulating regular physical activity as a means of health promotion involves the engagement of the PE professional as a player in the process. 

Hence, if the goal of PE is to promote health in a broad sense, the pedagogical approach would expand the attitudinal and affective dimensions as it aims to install a culture of non-sedentary lifestyle, for lifelong commitment to physical activity. The curricular conception and structure require an approach compatible with that objective. On the other hand, the curriculum is not immune to the teacher, and vice versa, as much as the academic culture of this professional is long lasting. The consequence is that the PE teacher’s conceptions, attitudes and values regarding the role of PE are inseparable from their performance. Accordingly, we start from the assumption that the curriculum is not neutral [17] and will necessarily be influenced by the conceptions of those involved in the process [18].

Thus, the objective of the present work is to verify concepts and attitudes of PE professionals and undergraduate students, in order to verify how they assess their role in the task of health promotion. The hypothesis is that the concepts, values, and attitudes are mismatched with the role attributed to them as health promoters, and that this mismatch may be related to the academic culture more than national curricular orientation or health initiatives. 

## 2. Materials and Methods 

This was a cross-sectional study that followed the guidelines ‘Strengthening the Reporting of Observational Studies in Epidemiology” (STROBE), cross-sectional version [19], as well as the “Improving the Quality of Web Surveys: The Checklist for Reporting Results of Internet E-Surveys” (CHERRIES) [20]. This study was approved by the National Board of Ethics Committee (National Protocol Number, CAAE: 89234218.6.0000.5676).

### 2.1. Participants and Setting

A total of 942 participants comprised the sample. The sample power calculation considered a universe of 390,766 professionals registered in the Federal Council of Physical Education, whose adhesion is mandatory by law for professional practice. The population of undergraduate students was estimated to be about 185,000. Thus, a confidence level of 95% and margin of error of 5% was adopted for survey sample estimation [21]. Calculation was performed with G*Power [22] and provided a minimum number of 384 participants needed. 

The inclusion criteria were being 18 years old or older, and being a PE professional, graduate, or undergraduate student. 

According to CHERRIES, the survey was pretested with a pilot sample of 39 participants to check item clarity and time for completion. It was an open survey, and the initial contact with potential participants was made during two major congresses with international attendance, as well as internet contact through professional and personal communication or invitation, with no advertisement or mailing list. 

The survey was administered through a free platform (Google Docs), and the responses were automatically captured. Participation was voluntary without incentives, which are forbidden by Brazilian laws. Data was collected from October 2108 to June 2019. Items were not randomized for participants without adaptative questioning or statistical correction for items, and 10 items appeared per page for eight pages, with a possible review step at each stage. All fields were mandatory; in this sense, it was not possible to gather an incomplete questionnaire. View rate and participation rate were not retrieved.

### 2.2. Instruments

All questionnaires were produced ad-hoc for the present study. The items were constructed and checked by four researchers comprised of a physical education professional and teacher, who is also the coordinator of a graduation course in Physical Education and has a Masters Degree in Public Health; a psychologist with an MSc in psychometry and PhD in health psychology; a geneticist, who takes part in the data analysis; and a PhD in Science Education and post-doc in Medical Education, and the Principal Investigator (P.I.) of the present work. The construction of items, as well as their validity and reliability, were performed according to the proposal by Lynn [23]. The questionnaires were as follows.

There was a sociodemographic questionnaire to characterize the participants, encompassing questions about sex, academic and professional background, etc.

Another questionnaire was on the role of school physical education, which aimed to verify the participant’s agreement regarding aspects of school PE. It contains 20 statements, divided into five domains with four items in each domain, and a three-level, Likert-type response scale: agree, indifferent, and disagree [24]. The domains contained statements about (1) PE as a means of health promotion, (2) the contents covered in the PE class, (3) the importance of PE in the school curriculum structure, (4) PE as a means of transmitting values, and (5) the purpose of school PE. In addition to thematic coherence, we also sought to produce items with some similarity, in order to verify the consistency in the responses given by the participants [25]. They were presented in a random order to the participants and reorganized for analysis and presentation. 

The third questionnaire was on the origin of the concepts and principles that determine the choices of the contents of a PE class. It contained six items, with a three-level, Likert-type response scale (agree, indifferent, and disagree) [24], which mention sources of knowledge that participants should refer to as potential reference points for professional practice. The items are presented in an gradient that starts from organized knowledge endorsed by educational guidelines, going to knowledge without scientific support or in accordance with specific guidelines [26].

The next questionnaire was on the attribution of responsibility for childhood obesity; it had 10 items with three levels of response, which were designed to consider social factors inside and outside the school environment. The items referring to the school context were related to the scope of PE. The items were presented at random to the participants [26].

The last questionnaire, which focused on aspects that motivate someone to practice physical activity, had five items with three levels of response, and the participants had to refer to motivating agents for an individual to make a regular practice of physical activity. The items were designed and ordered in a gradient, starting from extrinsic social to intrinsic elements, and were presented without specific ordering.

The results of the questionnaires were ordered by decreasing degree of agreement, due to the fact that the participant could attribute value to each item without any limitation. This is valid except for the sociodemographic and PE role assignment questionnaires. We assume that the highest proportion of agreement indicates a higher valuation or weight attributed to the item [25].

### 2.3. Data Analysis

The analysis was carried out both for the characterization of the answers and for the characterization of the respondents’ profile, according to the answers given. The data were extracted from Google Forms platform and exported to Microsoft Excel format. The coding was verified by two independent researchers, all data were correct and according to the pre-established format for each instrument. The database was then exported to SPSS 20 software (IBM, New York, NY, USA) for Macintosh, where statistical analyzes were performed. Descriptive analyses (frequency, percentage, average, and standard deviation) were performed to characterize the participants. The chi-square test was used to compare categorical variables between groups (student and professional; female and male). Continuous variables had their normality tested by the Kolmogorov-Smirnov test. We chose non-parametric tests and Mann-Whitney U to compare continuous variables by groups (student and professional; female and male). Spearman correlations were performed between continuous variables.

## 3. Results

This study involved a total of 942 participants. The average age of the participants was 28.15 (±10.4) years, with 63.4% working directly in the area of physical education. Of the professionals, 48.1% (177) worked in school physical education.

The sample characterization data are shown in Table 1.

Similarities and differences in the response patterns between the groups were verified through chi-squared tests. No differences were observed between professional male and female, as well as male and female students. Significant difference was observed for the aggregated data of professionals versus undergraduate students. Thus, all data from the questionnaires were presented separately for these groups, and the difference between the groups being tested for each of the items presented.

The questionnaire about the role of the school PE is described in the original order in which it was presented to the participants (Table 2).

The data about the origin of the concepts and principles that determine the choice of content for a physical education class is presented in Table 3.

Initial academic training is ranked first, followed by independent continuing education, chosen by the individual, then the individuals’ own conceptions, and in fourth place is the continuing education offered by the school and departments, which theoretically would be more aligned with the concepts defined by government documents; last is the media as an influencer in defining the content of their classes. Data regarding the attribution of responsibility for childhood obesity is presented in Table 4.

The survey is based on the principle that PE is an instrument for health promotion in a biomedical perspective, as it assumes that scholar PE may play a role in prevention or intervention towards child obesity. The first three positions reveal that both professionals and students attribute responsibility to child obesity to external agents regarding the school. The fourth position is occupied by a justification of the limitations of the reach of PE, followed by structural elements of professional development (5 and 6), then social and structural components of the scholar environment (7 and 8), the government (9), and in notably lower proportion, the PE teacher (10). 

The data about the valuing of aspects that motivates the individual in the practice of physical activity is presented in Table 5.

The participant positively values the statement about their role as motivators for the practice of physical activity (86.4% agree), but this weight is much lower (54.4% disagree) regarding the responsibility for childhood obesity (Table 4). However, in opposition, almost 45% of the participants attribute themselves as responsible for the childhood obesity. This data is coherent with the proportion of professionals that is involved with the school PE (48.1%).

Similarly, the influence of the family in childhood obesity is highly valued, as is the opposite regarding their influence as motivators for the practice of physical activity.

According to the participants, the media is less influential in the motivation of physical activity than in responsibility for childhood obesity. This is curious, since the demands of beauty, self-image, and fitness are very pervasive in the media messages.

Finally, both groups value the statement that the motivation for physical activity must be intrinsic and is related to health more. However, this attenuates the role of PE, since the motivation must come from the individual. In addition, it is associated with a concept of non-disease, due to the value attributed to a medical guidance, widely admitted as mandatory, posing this valuation as more related to the biomedical concept of PE.

## 4. Discussion

This study presents data from PE professionals and undergraduate students, where they regard themselves as key players on school health promotion. However, some findings suggest potential mismatches among what they value about procedures, attitudes or strategies, the role of PE in different settings, and what is proposed by national agencies of health and education.

In Brazil, the physical educator is recognized as a health professional, according to the National Curricular Parameters of Higher Education from the Ministry of Education, and by the Ministry of Health. Moreover, the National Curricular Parameters for school PE, defined by the Ministry of Education, mentions that the school PE aims for the adoption of healthy habits; the development of a responsibility with one’s own health and public health; the culture of movement and its affective domains; the promotion, maintenance, and recovery of health, leisure, and recreational physical activity; and the critical analysis of social values. However, the concept of health is not defined, and the word “health” is mentioned in context five times out of 22 occurrences of the word in a 96-page document [27]. 

A graduation course in PE is divided into two modalities: bachelor’s and licensure degrees. Each modality has its exclusive and non-interchangeable professional attributions. The bachelor performs the activity related to training, exercise, fitness, gym, and personal trainer, but is not allowed to work in school PE. The licensure degree professional carries out the activities related to school PE, from kindergarten to high school. With slight variations, the undergraduate course is divided in two parts: a basic cycle, where a set of disciplines are taken together by all students, and a specialty cycle, where each modality focuses on its specific content. Also, with variations, the bachelor’s curriculum is mainly based on biological, exercise, and training contents, and licensure curriculum is based on pedagogy and education backgrounds. Despite the different roles and areas of expertise, both are recognized as health professionals, and are assigned as agents of health promotion [10].

A datum that appears in the sociodemographic survey is that 66% of professionals and 82.9% of students surveyed do not know one of the main government programs, the “Health in the School” program. However, the role of school PE and the PE teacher is not completely clear in either the national curricular directives or in the “Health in the School” program. Moreover, these documents do not define or adopt a clear concept of “health” or “health promotion”. Such structural mismatches leads to a dissipation of efforts and goes contrary to several countries [28], even in Latin America [29]. 

Regarding the average professional qualification time (11.33 ± 10.2 years), it is possible to suggest that some of these professionals were not aware of the program during their training. Although the weekly workload is high (34.99 ± 25.71 hours), more than 70% of professionals took courses after graduation, and could have had some contact with the “Health in the School” program. This data raises concern when considering the undergraduate student population. Since the launch of the initiative 13 years ago, there has been time to incorporate it into the curriculum components. Part of the universe of students surveyed has already completed the initial periods (4.76 ± 2.37 semesters) and could have received part of this content. This data suggests a possible mismatch between academic training, curriculum guidelines, and health promotion policies.

On the other hand, the data that emerge from the other questionnaires suggest that the perception and appreciation of the role of PE are more aligned with health promotion, even though this role is not entirely clear to professionals and students. Considering that PE classes are a legitimate setting for the education and training of adolescents, the values and attitudes of PE teachers towards the role of school PE and its relation to health are of paramount importance [28].

### 4.1. The Role of School Physical Education

The literature discusses how PE contributes to health, although there is no consensus on how to achieve its goals, or what parameters would indicate its success [2]. Our data show that although the role of school PE as a means of promoting health is valued, professionals and undergraduate students do not attribute the responsibility for childhood obesity to PE, or believe that it encourages the practice of physical activity. These data is also in agreement with authors who state that the aims of the PE remain under debate [30]; therefore, in the absence of consensus, several meanings will flourish. This allows the valuation of several outcomes, such as physical and social skills, moral values, spirituality, intellectual ability, health (including obesity), fitness, or recreation. 

This issue is illustrated by the variety of uses or strategic purposes attributed to PE in different countries. The role of school PE in the United States is related to the SPARK/HOPE principles (Sports, Play, and Active Recreation for Kids/Health Opportunities through Physical Education) [6]; in the United Kingdom, a sports-centered role has also been considered [31], passing through several health-centered roles in a broader conception, such as in Canada [32], Denmark [1], Spain [33], Greece [34], South Korea, Hong Kong, and China [35], and considering a deeper physical activity literacy in the curriculum, such as in Austria [36] and Wales [37], to countries that incorporated the term “health” and are now referring to it as “physical education and health”, such as in Sweden [38], or “health and physical education” in Australia. These initiatives reinforce that PE goes beyond sports, and health is even more emphasized in the curriculum [4]. The aim is the adoption of healthy living habits from school through the influence of PE classes [16].

However, the concept of health is broad, as well as what should be the role of PE in health promotion. A wide range of possibilities arises from the potential attributions for PE as a means for health promotion, and are a topic of vigorous discussion [39]. Even so, it is undeniable that PE has the potential to play a strategic role in health promotion by tradition and vocation, despite the absence of a consensus about the role of PE as a tool for health promotion, which concept of health and physical activity is adopted, and by which means these goals could be achieved [40].

Our data suggests that the school PE is not relevant to entering college, according to both professionals and undergraduate students. This is aligned with a concept of curriculum strictly centered on the cognitive domain, even though it is an audience of teachers and PE students who supposedly should value affective and motor domains. This possibly goes back to the fact that PE has a curriculum historically focused on the use of sports as a substrate [41]. This conception is corroborated by data indicating that school PE teaches how to compete. The agreement at around 80% suggests a very widespread concept among this audience. Therefore, if teaching how to compete is an assignment of school PE, then initiatives to use sport as a means to promote physical activity should not be considered as a minor approach in contexts where fewer alternatives are available [42]. On the other hand, this may suggest a conflict with the salutogenic meaning of PE, where competition is not an aspect to be necessarily valued.

Not surprisingly, the group of questions that verify the value attributed to the importance of school PE in the school context or school curriculum are very emphatic and coherent. The group who disagree with this fact, which varies from 5% to 12% of the respondents, is mostly composed of participants who do not work in the PE area, despite their academic background.

Data regarding content delivery in PE shows that the groups are divided, with 52.8% agreeing that it should be different. These data need to be carefully interpreted, as they do not inform about which form should be used. On the other hand, the other items in this subgroup suggest that the participants are not frankly aligned with the way in which the content is transmitted.

Regarding time dedicated to health promotion at school, the coherence in answers suggests that the participants share views on the item that is consistent with the literature. In addition, this view is also consistent with the valuing of health as a goal of PE. It also suggests that conceptions about sport and physical activity have no significant differences between men and women. This meets a previous demand for greater female participation in the EP [43], given that it is corroborated by the proportion of participants in this research. 

### 4.2. What Determine the Choices of the Contents of the Physical Education Class

Our data shows that what determines the choices of the contents of the PE class is mainly based on the acquired knowledge from graduation, followed by independent continuing education, as well as by self-concepts. Although the last two are not necessarily aligned with current guidelines, surprisingly the participants value the self-concepts, which are a questionable source of knowledge to say the least. Nevertheless, this reinforces the hypothesis of the present work that the values of the professionals and students put a considerable weight in the definition of PE class. Indeed, this observation is supported by previous research showing that the teachers’ content knowledge affects the students’ achievements of learning outcomes [44]. In this case, the teachers’ most valued source of content is self-referent, obsolete, or based on questionable sources.

On the other hand, it has been suggested that the PE teacher is not receiving adequate tools to face contemporary challenges in the school environment [45]. This can be observed in many items of the present study, where PE professionals and students blame the lack of continuing education opportunities.

### 4.3. The Attribution of Responsibility for Childhood Obesity

This mismatch can influence the results of school PE, which corroborates the data on obesity and physical activity at school reported in data from a national survey. The National Student Health Survey had, among its several objectives, monitoring risk factors and health protection for Brazilian students and identifying priority issues for the development of public policies aimed at promoting the health of adolescents [11,46]. They indicated overweightness in almost 30% of students in schools in the most developed regions, and obesity in more than 10% of students [11,46]. Most adolescents (60.8%) were classified as insufficiently active, and 4.8% as inactive; in addition, more than two PE classes were available in less than 48% of the schools, with 50% in public schools and less than 40% in private schools [11,46].

However, data on the practice of physical activity may not necessarily be related to the perception of health, but rather to impositions regarding body image and the desire to lose weight. Almost 40% of students resort to practices that are harmful to their health. Aggregate data revealed that around 6% of students reported inducing vomiting or taking laxatives as a means of losing weight or avoiding gaining weight. Ingestion of drugs, formulas, or some product to lose weight without medical supervision was more common among boys (6.8%) than among girls (5.2%) [11,46]. These data also reinforce our finding about the influence of the media attributed by PE staff, with a more radical view of the problem of obesity. Other authors also questions the existence of a global issue regarding obesity [30].

These findings indicate that the approaches to health in school PE are insufficient, or at least that the school PE is not meeting objectives in health promotion. In this sense, regardless of the PE concept adopted, indicators of child health are matter of concern. 

Our data shows that the participants agree with the statement that if more time were available for the PE class, it would be possible to act against childhood obesity in the school context. Even though the weight attributed to the items suggests a major role of external factors to PE, a role in health promotion for PE is observed here.

Our data on the perception that respondents have about the responsibility for childhood obesity are aligned with time spent with technologies, and also on the role of the family, since this is a domestic regulation that is part of family habits and culture. This perception is consistent with national data that showed that 56.1% of students spend more than three hours a day in front of games, computers, or other activities that characterize physical inactivity [11,46], which is less than that observed in other studies, where the levels of physical activity were considered satisfactory for 25.0% of the 11-year-olds and 16.0% of the 15-year-olds [47]. 

### 4.4. Aspects that Motivate Someone to Practice Physical Activity

Another fact that has emerged from our work is that the importance attributed to school PE by professionals and students is not consistent with data on student attendance in classes and adherence to activities. Physical education class evaluations have shown low student participation and increased dropout [48].

Our findings show that both PE professionals and undergraduate students mainly attribute intrinsic motivations for individuals to perform physical activity. This data is in agreement with the data of a wide systematic review that included more than 60,000 subjects from 10 to 90 years old in different regions of Brazil to characterize the barriers to physical activity [49]. The authors of this study report several reasons, with an emphasis on lack of support from family, lack of motivation or companionship, inappropriate climate, lack of facilities, and laziness. In lower proportions also appear lack of skills for physical activity, lack of knowledge about physical activity, and preference for other activities.

However, it must be considered that the lack of infrastructure and support from the school community were also considered relevant in the present study. This perception is in line with national data that 7 out of 10 students (72.8%) attend schools with sports courts, and 92% have access to sports materials in conditions of use. There is a notable difference between the public and private schools, where there is a sports court in 69.2% and 94.1% of schools respectively, and changing rooms in 22.2% of the public network but 67.5% of the private network [11,46]. 

Moreover, disengagement is associated with other factors, and is also reported in countries with more favorable conditions [50]. Even so, these aspects can interfere with the teacher’s activity, since teacher’s attitudes and behaviors influence the student’s behavior, with technical knowledge about the content not being sufficient for their activity [51].

Our data show that both professionals and undergraduate students value factors outside the school in all questionnaires. This data is consistent with previous findings that state that the school PE teacher has a limited impact on decisions about the role of school PE and how it should be taught, mainly due to recurring assumptions with origins traced from undergraduate studies [38]. Also, it is presented here that surveyed professionals and undergraduate students value a role in promoting fitness-centered health for physical activity. On the other hand, a limited reach in the implementation of these conceptions is attributed to factors external to the school environment (family and media), as intrinsic factors of the motivations for physical activity do not consider PE as a determinant.

Finally, our data shows that regulations must be more aligned with the PE teacher training, in agreement with recent studies that claim to support schools developing a healthy environment, clear regulations, improvement of physical spaces for physical activity, and understanding the challenges posed to the school PE teachers [10].

Despite the lack of studies verifying the impact of the school in health promotion in Brazil [10], and the need for data from the non-English speaking world [33], our study shows that despite the efforts and laws regarding the role of the physical educator as a health profession, neither undergraduate students nor professionals’ values encompass a paradigm shift towards health promotion. On the other hand, values regarding school PE among men and women were similar, suggesting a common view about the role of school PE. This may be considered a positive aspect, since the values of physical education should be beyond gender differences.

Also, we found that academic values (or academic curricula) are not necessarily aligned with the expected role of health promotion proposed by national agencies. In this case, the values and attitudes reported by PE professionals and students possibly originate in the undergraduate period. In this sense, the academic culture is prominently valued in the long term, and is overshadowing possible organized efforts to establish the school PE teacher or the PE professional as a player in health promotion. Also, there is uncertainty concerning the role of school PE, as a high value is attributed to external factors such as family, technology, and media as being responsible for childhood obesity and motivation for physical activity.

### 4.5. Strenghts

Our study was comprised of 368 professionals and 574 undergraduate students, which comprises adequate sample power. Also, the absence of significant differences between male and female participants concerning the items surveyed suggests a cohesion in concepts that is important to help fill the gap of gender differences historically reported in the field.

### 4.6. Limitations

Despite the sample size and regional distribution of this sample, our study is restricted to a Brazilian sample. However, data about childhood health is of growing concern, and the number of PE professionals is still growing. In this sense, to define the role of these professionals in public health is of renewed interest. Lastly, our study is based on a self-reported survey.

## 5. Conclusions

At the same time when school PE is regarded as a player in health promotion by PE professionals and students, our data suggests that concepts of health and health promotion are not clear with regard to in which manner the school PE and its curriculum should be designed to serve as an instrument of health promotion. 

Although there are strong arguments about the weakened initial formation of the PE professional, current social issues (technology, violence, urbanization, and reduction of spaces for physical activity) are legitimate justifications for the scenario raised here. National issues also permeate the discussion due to the lack of structure and material for professional continuing education, as well as student families as an important source of misconceptions, which do not encourage an active and healthy lifestyle, and school communities that do not understand physical education as an “important discipline”. The mismatch between academic training and academic culture represented by the values and attitudes declared by PE professionals and undergraduate students reinforces the need for alignment of purposes in the training of professionals and in the clear attribution of the role of school PE in the current needs of public health.

## Figures and Tables

**Table 1:** Sociodemographic characteristic of the participants.

Characteristic		Professional *n* (%)	Student *n* (%)
Sex	Female	154 (41.8)	264 (46.0)
	Male	214 (58.2)	310 (54.0)
Graduate studies performed at	Public, county	8 (2.2)	34 (5.9)
	Public, state	42 (11.4)	12 (2.1)
	Public, federal	61 (16.6)	15 (2.6)
	Communitary	44 (12.0)	162 (28.2)
	Private	213 (57.9)	351 (61.0)
Highest degree	Graduation	110 (29.9)	-
	Specialist	187 (50.8)	-
	Master’s degree	50 (13.6)	-
	PhD	21 (5.7)	-
Specialty	Bachelor	47 (12.8)	316 (55.1)
	Graduate	89 (24.2)	243 (42.3)
	Bachelor and graduate	232 (63.0)	15 (2.6)
Professional activity in physical education (PE)	No	41 (11.1)	204 (53.0)
	Yes	327 (88.9)	270 (47.0)
Job sector	Public	163 (44.3)	
	Private	98 (26.6)	
	Public and private	58 (15.8)	
	Freelance	49 (13.3)	
Work in school PE	No	191 (51.9)	
	Yes	177 (48.1)	
Know the program “Health in School”	No	243 (66.0)	476 (82.9)
	Yes	125 (34.0)	98 (17.1)
		**Mean (SD)**	**Mean (SD)**
Age		35.89 (10.78)	23.18 (6.33)
Semesters studied	-	-	4.76 (2.37)
Years active	-	11.72 (10.31)	-
Years since graduation	-	11.33 (10.20)	-
Weekly working hours	-	34.99 (15.71)	-
How many jobs	-	1.55 (0.81)	-

SD: Standard deviation.

**Table 2:** Questionnaire on the role of school physical education (PE).

Affirmatives		Professional (*n* = 368)	Students (*n* = 574)	*p*	Total (*n* = 942)
		*n* (%)	*n* (%)		*n* (%)
Physical education:					
1. Promote health	**A**	**309 (84.0)**	**510 (88.9)**	0.071	**819 (86.9)**
	I	27 (7.3)	25 (4.4)		52 (5.5)
	D	23 (8.7)	39 (6.8)		71 (7.5)
2. Has no subject to study	**A**	**25 (6.8)**	**36 (6.3)**	0.944	**61 (6.5)**
	I	23 (6.2)	35 (6.1)		58 (6.2)
	D	320 (87.0)	503 (87.6)		823 (87.4)
3. Stimulates the habit of physical education	**A**	**324 (88.0)**	**534 (93.0)**	0.031 *	**858 (91.1)**
	I	32 (8.7)	30 95.2)		62 (6.6)
	D	12 (3.3)	10 (1.7)		22 (2.3)
4. Has little importance in the school curriculum	**A**	18 (4.9)	44 (7.7)	0.120	62 (6.6)
	I	11 (3.0)	10 (1.7)		21 (2.2)
	D	339 (92.1)	520 (90.6)		859 (91.2)
5. Is not important to enter college	**A**	57 (15.5)	102 (17.8)	0.656	159 (16.9)
	I	61 (16.6)	94 (16.4)		155 (16.5)
	D	250 (67.9)	378 (65.9)		628 (66.7)
6. Teaches how to compete	**A**	290 (78.8)	481 (83.8)	0.021 *	771 (81.8)
	I	47 (12.8)	69 (12.0)		116 (12.3)
	D	31 (8.4)	24 (4.2)		55 (5.8)
7. Would have its time better used in other disciplines	**A**	16 (4.3)	56 (9.8)	<0.001 *	72 (7.6)
	I	13 (3.5)	47 (8.2)		60 (6.4)
	D	339 (92.1)	471 (82.1)		810 (86.0)
8. Is less important than other disciplines	**A**	12 (3.3)	24 (4.2)	0.017 *	36 (3.8)
	I	16 (4.3)	52 (9.1)		68 (7.2)
	D	340 (92.4)	498 (86.8)		838 (89.0)
9. Teaches to work in a group	**A**	347 (94.3)	555 (96.7)	0.205	902 (95.8)
	I	11 (3.0)	10 (1.7)		21 (2.2)
	D	10 (2.7)	9 (1.6)		19 (2.0)
10. Taught me few things	**A**	39 (10.6)	93 (16.2)	0.007 *	132 (14.0)
	I	38 (10.3)	80 (13.9)		118 (12.5)
	D	291 (79.1)	401 (69.9)		692 (73.5)
11. Should be taught in a different manner	**A**	209 (56.8)	288 (50.2)	0.124	497 (52.8)
	I	85 (23.1)	146 (25.4)		231 (24.5)
	D	74 (20.1)	140 (24.4)		210 (22.7)
12. Has not enough time to promote health	**A**	92 (25.0)	118 (20.6)	0.270	210 (22.3)
	I	53 (14.4)	91 (15.9)		144 (15.3)
	D	223 (60.6)	365 (63.6)		588 (62.4)
13. Does not teach useful things	**A**	23 (6.2)	65 (11.3)	0.002 *	88 (9.3)
	I	32 (8.7)	75 (13.1)		107 (11.4)
	D	313 (85.1)	434 (75.6)		747 (79.3)
14. Serves only to ball-playing	**A**	5 (1.4)	12 (2.1)	0.265	17 (1.8)
	I	9 (2.4)	7 (1.2)		16 (1.7)
	D	354 (96.2)	555 (96.7)		909 (96.5)
15. Could have other disciplines instead	**A**	4 (1.1)	14 (2.4)	0.295	18 (1.9)
	I	9 (2.4)	17 (3.0)		26 (2.8)
	D	355 (96.5)	543 (94.6)		898 (95.3)
16. Is important for my formative period	**A**	330 (89.7)	470 (81.9)	0.005 *	800 (84.9)
	I	25 (6.8)	69 (12.0)		94 (10.0)
	D	13 (3.5)	35 (6.1)		48 (5.1)
17. Helps train future athletes	**A**	280 (76.1)	489 (85.2)	<0.001 *	759 (81.6)
	I	45 (12.2)	59 (10.3)		104 (11.0)
	D	43 (11.7)	26 (4.5)		69 (7.3)
18. Is a time for relaxation	**A**	213 (57.9)	347 (60.5)	0.726	560 (59.4)
	I	75 (20.4)	108 (18.8)		183 (19.4)
	D	80 (21.7)	119 (20.7)		199 (21.1)
19. Need more time to promote health	**A**	265 (72.0)	455 (79.4)	0.023 *	720 (76.4)
	I	61 (16.6)	78 (13.6)		139 (14.8)
	D	42 (11.4)	41 (7.1)		83 (8.8)
20. Conveys values about healthy competitiveness	**A**	322 (87.5)	522 (90.9)	0.223	844 (89.6)
	I	32 (8.7)	38 (6.6)		70 (7.4)
	D	14 (3.8)	14 (2.4)		28 (3.0)

D: disagree; I: indifferent; A: agree. Between groups, comparison performed through chi-squared test, with *p*-values indicated with (*) for difference between groups.

**Table 3:** Questionnaire on the origin of the concepts and principles that determine the choices of content for a physical education class.

What Determines the Content:		Professional (*n* = 368)	Student (*n* = 574)	Rank (S, P)	*p*	Total (*n* = 942)
Academic training only	**A**	**312 (84.8)**	**479 (83.4)**	1, 1	0.017 *	**791 (84.0)**
	I	40 (10.9)	85 (14.8)			125 (13.3)
	D	16 (4.3)	10 (1.7)			26 (2.8)
Independent continuing education	**A**	**287 (78.0)**	**436 (76.0)**	2, 2	0.230	**723 (76.8)**
	I	59 (16.0)	113 (19.7)			172 (18.3)
	D	22 (6.0)	25 (4.4)			47 (5.0)
Self concepts	**A**	**263 (71.5)**	**390 (67.9)**	4, 3	0.463	**653 (69.3)**
	I	76 (20.7)	138 (24.0)			214 (22.7)
	D	29 (7.9)	46 (8.0)			75 (8.0)
Government- and school-provided continuing education	**A**	**251 (68.2)**	**420 (73.2)**	3, 4	0.002 *	**671 (71.2)**
	I	79 (21.5)	129 (22.5)			208 (22.1)
	D	38 (10.3)	25 (4.4)			63 (6.7)
Specialized media	**A**	**195 (53.0)**	**322 (56.1)**	5, 5	0.037 *	**517 (54.9)**
	I	112 (30.4)	190 (33.1)			302 (32.1)
	D	61 (16.6)	62 (10.8)			123 (13.1)
General media	**A**	**153 (41.6)**	**286 (49.8)**	6, 6	0.003 *	**439 (46.6)**
	I	132 (35.9)	205 (35.7)			337 (35.8)
	D	83 (22.6)	83 (14.5)			166 (17.6)

D: disagree; I: indifferent; A: agree. Between-group comparison performed through a chi-squared test, with *p*-values identified with (*) for statistical difference between groups. Items were presented to the participants in the order of the first column on the left. The column “rank” presents the order of importance attributed to each item by students (S) and professionals (P), respectively (S, P).

**Table 4:** Questionnaire on the attribution of responsibility for childhood obesity.

Who Is Responsible for Childhood Obesity		Professional (*n* = 368)	Student (*n* = 574)	*p*	Rank (S, P)	Total (*n* = 942)
Family	**A**	**320 (87.0)**	**478 (83.3)**	0.299	2, 1	**798 (84.7)**
	I	36 (9.8)	70 (12.2)			106 (11.3)
	D	12 (3.3)	26 (4.5)			38 (4.0)
Technology	**A**	**293 (79.6)**	**490 (85.4)**	0.066	1, 2	**783 (83.1)**
	I	47 (12.8)	50 (8.7)			97 (10.3)
	D	28 (7.6)	34 (5.9)			62 (6.6)
Media	**A**	**264 (71.7)**	**420 (73.2)**	0.126	3, 3	**684 (72.6)**
	I	52 (14.1)	96 (16.7)			148 (15.7)
	D	42 (14.1)	58 (10.1)			110 (11.7)
Short weekly class time to generate results	**A**	**240 (65.2)**	**383 (66.7)**	0.819	4, 4	**623 (66.1)**
	I	64 (17.4)	100 (17.4)			164 (17.4)
	D	64 (17.4)	91 (15.9)			155 (16.5)
Deficiencies in graduation	**A**	**194 (52.7)**	**315 (54.9)**	0.614	5, 5	**509 (54.0)**
	I	99 (26.9)	138 (24.0)			237 (25.2)
	D	75 (20.4)	121 (21.1)			196 (20.8)
Lack of continuing education for teachers	**A**	**188 (51.1)**	**219 (38.2)**	<0.001	9, 6	**407 (43.2)**
	I	74 (20.1)	140 (24.4)			214 (22.7)
	D	106 (28.8)	215 (37.5)			321 (34.1)
Lack of infrastructure for PE classes	**A**	**186 (50.5)**	**297 (51.7)**	0.489	6, 9	**483 (51.3)**
	I	73 (19.8)	126 (22.0)			199 (21.1)
	D	109 (26.3)	151 (29.6)			260 (27.6)
School community	**A**	**179 (48.6)**	**294 (51.2)**	0.486	7, 7	**473 (50.2)**
	I	120 (32.6)	166 (28.9)			286 (30.4)
	D	69 (18.8)	114 (19.9)			183 (19.4)
Government	**A**	**168 (45.7)**	**259 (45.1)**	0.875	8, 8	**427 (45.3)**
	I	113 (30.7)	171 (29.8)			284 (30.1)
	D	87 (23.6)	144 (25.1)			231 (24.4)
The school PE teacher	**A**	**95 (25.8)**	**164 (28.6)**	0.633	10, 10	**259 (27.5)**
	I	67 (18.2)	104 (18.1)			171 (18.2)
	D	206 (56.0)	306 (53.3)			512 (54.4)

D: disagree; I: indifferent; A: agree. Between-group comparison performed through chi-squared test, with *p*-values indicated with (*) for difference between groups. Items were presented in random order to the participants. The column with rank values are related to the order of importance attributed to each item by students (S) and professionals (P), respectively.

**Table 5:** Questionnaire of aspects that motivate the practice of physical activity.

Motivation for Practicing Physical Activity		Professional (*n* = 368)	Student (*n* = 574)	Rank (S, P)	*p*	Total (*n* = 942)
Concern for one’s own health	**A**	**319 (86.7)**	**524 (91.3)**	1, 1	0.070	**843 (89.5)**
	I	34 (9.2)	37 (6.4)			71 (7.5)
	D	15 (4.1)	13 (2.3)			28 (3.0)
The school PE teacher	**A**	**315 (85.6)**	**499 (86.9)**	3, 2	0.484	**814 (86.4)**
	I	39 (10.6)	61 (10.6)			100 (10.6)
	D	14 (3.8)	14 (2.4)			28 (3.0)
Medical guidance	**A**	**312 (84.8)**	**517 (90.1)**	2, 3	0.052	**829 (88.0)**
	I	40 (10.9)	42 (7.3)			82 (8.7)
	D	16 (4.3)	15 (2.6)			31 (3.3)
Family	**A**	**311 (84.5)**	**471 (82.1)**	4, 4	0.467	**782 (83.0)**
	I	40 (10.9)	78 (13.6)			118 (12.5)
	D	17 (4.6)	25 (4.4)			42 (4.5)
Media	**A**	**221 (60.1)**	**317 (55.2)**	5, 5	0.307	**538 (57.1)**
	I	81 (22.0)	148 (25.8)			229 (24.3)
	D	66 (17.9)	109 (19.0)			175 (18.6)

D: disagree; I: indifferent; A: agree. Between-group comparison performed through chi-squared test, with *p*-values indicated with (*) for difference between groups. Items were presented in random order to the participants. The column with rank values are related to the order of importance attributed to each item by students (S) and professionals (P), respectively.
